# CAR T-Cells Targeting the Integrin αvβ6 and Co-Expressing the Chemokine Receptor CXCR2 Demonstrate Enhanced Homing and Efficacy against Several Solid Malignancies

**DOI:** 10.3390/cancers11050674

**Published:** 2019-05-14

**Authors:** Lynsey M. Whilding, Leena Halim, Benjamin Draper, Ana C. Parente-Pereira, Tomasz Zabinski, David Marc Davies, John Maher

**Affiliations:** 1 King’s College London, School of Cancer and Pharmaceutical Studies, Guy’s Hospital, Great Maze Pond, London SE1 9RT, UK; lynsey.whilding@kcl.ac.uk (L.M.W.); Leena.halim@kcl.ac.uk (L.H.); benjamin.draper@kcl.ac.uk (B.D.); anacatpp@gmail.com (A.C.P.-P.); tomasz.zabinski@kcl.ac.uk (T.Z.); david.m.davies@kcl.ac.uk (D.M.D.); 2 Department of Clinical Immunology and Allergy, King’s College Hospital NHS Foundation Trust, London SE5 9RS, UK; 3 Department of Immunology, Eastbourne Hospital, East Sussex BN21 2UD, UK

**Keywords:** CAR-T, immunotherapy, αvβ6, integrin, homing, chemokine receptor, solid tumor, cancer, chimeric antigen receptor, T-cell

## Abstract

Despite the unprecedented clinical success of chimeric antigen receptors (CAR) T-cells against haematological malignancy, solid tumors impose a far greater challenge to success. Largely, this stems from an inadequate capacity of CAR T-cells that can traffic and maintain function within a hostile microenvironment. To enhance tumor-directed T-cell trafficking, we have engineered CAR T-cells to acquire heightened responsiveness to interleukin (IL)-8. Circulating IL-8 levels correlate with disease burden and prognosis in multiple solid tumors in which it exerts diverse pathological functions including angiogenesis, support of cancer stem cell survival, and recruitment of immunosuppressive myeloid cells. To harness tumor-derived IL-8 for therapeutic benefit, we have co-expressed either of its cognate receptors (CXCR1 or CXCR2) in CAR T-cells that target the tumor-associated αvβ6 integrin. We demonstrate here that CXCR2-expressing CAR T-cells migrate more efficiently towards IL-8 and towards tumor conditioned media that contains this cytokine. As a result, these CAR T-cells elicit superior anti-tumor activity against established αvβ6-expressing ovarian or pancreatic tumor xenografts, with a more favorable toxicity profile. These data support the further engineering of CAR T-cells to acquire responsiveness to cancer-derived chemokines in order to improve their therapeutic activity against solid tumors.

## 1. Introduction

The epithelial-specific integrin αvβ6 is over-expressed in multiple solid tumors, including those derived from the pancreas, head and neck, breast, colon, stomach, lung, cervix, and fallopian tube/ovary [1,2,3,4,5,6,7,8]. Aberrant expression of αvβ6 by tumors correlates with poor prognosis, reflecting its role in promoting the activation of transforming growth factor (TGF)-β, tumor cell migration, invasion, and epithelial to mesenchymal transition [3,9]. In healthy tissues, αvβ6 is either undetectable or present at very low levels, while expression is physiologically upregulated during wound healing [10], lung injury, and fibrosis of liver, kidney, and other organs [11]. Conceptually, these attributes render αvβ6 an appealing target both for cancer imaging, for example using radiolabeled peptides [12], and for therapeutic intervention using monoclonal antibodies [3,13] and chimeric antigen receptor (CAR) T-cells [14,15]. 

CAR T-cell immunotherapy has achieved unprecedented benefit in patients with CD19-positive leukaemia or lymphoma [16,17,18,19,20,21], culminating in the widespread regulatory approval of Yescarta (axicabtagene ciloleucel) and Kymriah (tisagenlecleucel) for these indications. However, in contrast, most patients with solid tumors have experienced negligible benefits from this emerging therapeutic modality [22]. This reflects the myriad physical, molecular, and immunological obstacles deployed by solid tumors to ensure their self-preservation [22,23,24]. To address this impasse, much effort has been invested into the identification of more tumor-specific targets [25,26,27,28], use of regional CAR T-cell delivery systems [29] and the development of strategies to enhance the homing [30,31], penetration [32], and functional persistence of these cells within malignant deposits [33,34].

Tumor cells characteristically secrete a range of chemokines. In principle, these may be exploited to direct recruitment of CAR T-cells by co-expression of a suitably responsive chemokine receptor. Elevated serum levels of the pro-inflammatory chemokine interleukin (IL)-8 (CXCL8) are associated with disease burden and poor prognosis in multiple tumor types, including melanoma, renal cell carcinoma, non-small cell lung cancer (NSCLC), pancreatic, breast, and ovarian cancer [35,36]. Interleukin-8 acts as a chemoattractant for neutrophils and myeloid-derived suppressor cells and thereby contributes to the immunosuppressive nature of the tumor microenvironment. In addition, expression of IL-8 correlates with that of the αvβ6 integrin in colorectal and lung cancer disease states in which these two molecules interact to promote tumor cell proliferation and migration [37,38]. Interleukin-8 belongs to the cysteine-amino acid-cysteine (CXC) family of chemokines and, along with CXCL6, is a ligand for CXCR1. While IL-8 also binds to CXCR2, this receptor is more promiscuous and binds all chemokines (e.g., CXCL1-8) that contain both ELR and adjacent CXC motifs. 

We have previously described a CD28-containing second generation CAR targeted against αvβ6 using a 20mer peptide (A20FMDV2) derived from a foot and mouth disease virus capsid protein. These αvβ6 re-targeted CAR T-cells (A20-28z) caused regression of multiple solid tumor xenografts representative of pancreatic, breast, and ovarian cancer [15]. We hypothesized that co-expression of the IL-8 chemokine receptors CXCR1 or CXCR2 would enhance CAR T-cell migration towards tumor cells and thereby further improve therapeutic activity. Here, we demonstrate that IL-8 is produced by multiple αvβ6-positive tumor cell lines and is detected in the bloodstream of mice engrafted with five distinct tumor xenografts that express this integrin. Both CXCR1 and CXCR2-containing CAR T-cells demonstrated increased migration towards IL-8 and conditioned media that contained this chemokine. Moreover, T-cells that co-express the A20-28z CAR together with CXCR2 achieved improved tumor control in vivo compared to CAR T-cells that lack this chemokine receptor, without associated toxicity. 

## 2. Results

### 2.1. IL-8 Is Secreted by Multiple Tumor Cell Lines and Is Detectable in the Circulation of Tumor-Bearing Mice

**Figure 1:**
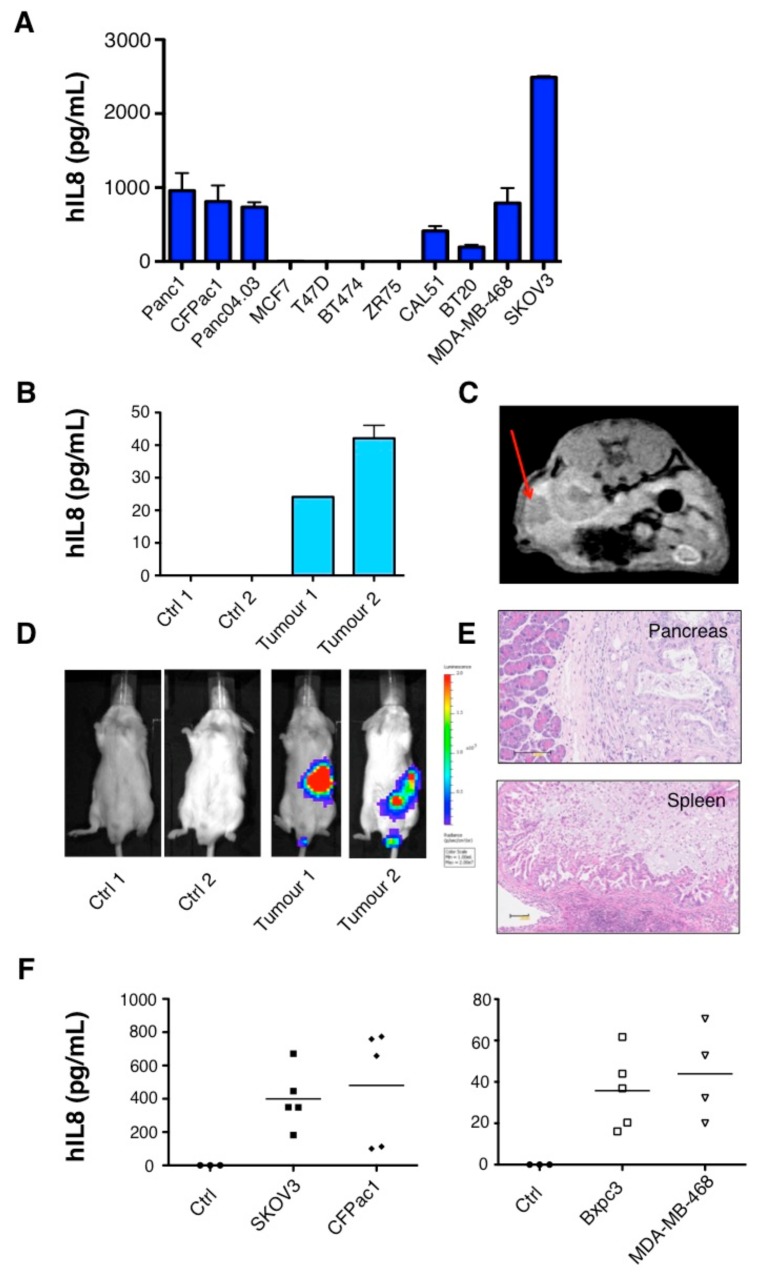
Detection of Interleukin-8 (IL-8) in the supernatant of cancer cell lines and in the circulation of mice with solid tumor xenografts. (**A**) Supernatant was collected from pancreatic, luminal, and triple negative breast or ovarian cancer cell lines 48 h after seeding 5 × 10^5^ cells in a 6-well plate. Data show the mean ± standard error of the mean (SEM) of IL-8 measured using enzyme-linked immunosorbent assay (ELISA) in 3–4 independent experiments. (**B**) Plasma levels of human IL-8 in mice bearing orthotopic Panc04.03 pancreatic xenografts as detected by (**C**) magnetic resonance imaging (MRI) and (**D**) bioluminescence imaging (BLI). Red arrow denotes location of tumor in MRI scan in (**C**). Control (Ctrl) mice were tumor-free. (**E**) Orthotopic tumor infiltration of pancreas and spleen was confirmed histologically by H and E staining of tissue. Scale bar: 100 µM. (**F**) Circulating human IL-8 was detected in the plasma of mice bearing intraperitoneal tumor xenografts of ovarian, pancreatic, and breast origin. Data show the mean and individual data points for each mouse.

To establish models of IL-8 producing cancer, we analyzed supernatants collected from a panel of pancreatic, breast, and ovarian cancer cell lines. High levels of IL-8 were produced by all three pancreatic cell lines tested (mean range 735–959 pg/mL), by SKOV3 ovarian cancer cells (mean 2492 pg/mL), and by the triple negative breast cancer (TNBC) cell lines CAL51, BT20, and MDA-MB-468 (mean range 197–790 pg/mL). In contrast, IL-8 was absent in supernatant derived from breast cancer cell lines of luminal subtype (Figure 1A). 

Next, we tested whether tumor cells secrete IL-8 when propagated as xenografts in immunocompromised SCID-Beige mice. Low levels of circulating human IL-8 were detected in two mice 46 days after Panc0403 cells were injected orthotopically into the pancreas (Figure 1B). Tumor engraftment was confirmed by magnetic resonance imaging (MRI) (Figure 1C), bioluminescent imaging (Figure 1D), and by subsequent histological analysis of the pancreas and spleen (Figure 1E). Similarly, IL-8 was detected in the plasma of all mice bearing four distinct intraperitoneal tumors of ovarian, pancreatic or TNBC origin (Figure 1F). As expected, human IL-8 was undetectable in non-tumor bearing mice (Figure 1B,F).

### 2.2. CAR T-Cells Targeted against αvβ6 Integrin That Co-Express CXCR1 or CXCR2 Retain Cytolytic Activity

**Figure 2:**
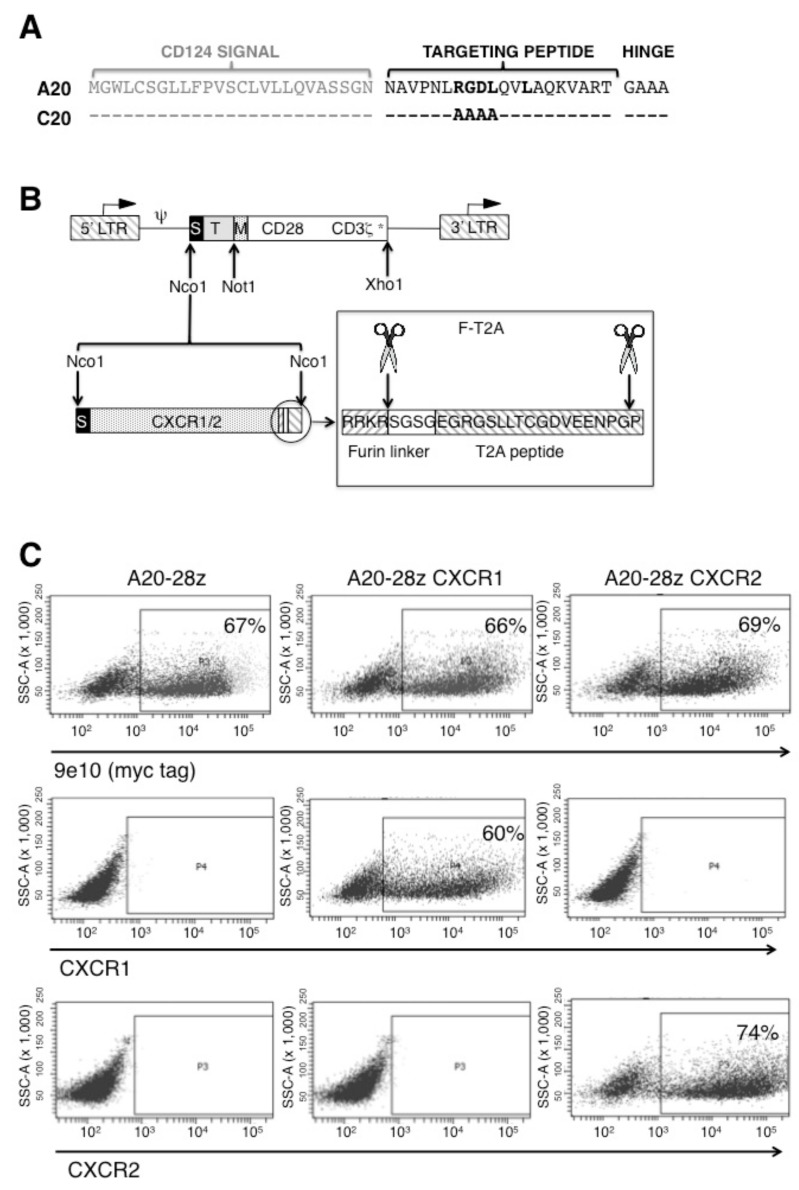
Design of retroviral constructs. (**A**) In order to engineer an αvβ6-targted chimeric antigen receptors (CAR), the A20 peptide derived from the capsid protein VP1 from Foot and Mouth Disease Virus (serotype 01 BFS) was placed downstream of a CD124 signal peptide. A matched but scrambled peptide (named C20) was generated in which RGDL was replaced with AAAA. (**B**) The SFG retroviral vector was used to express CARs in human T-cells. LTR: long terminal repeat; S: signal peptide; T: CAR targeting peptide; M: human c-myc epitope tag, recognized by 9e10 antibody. In some constructs, equimolar co-expression of the chemokine receptor CXCR1 or CXCR2 was achieved using a *Thosea Asigna* (T)2A ribosomal skip peptide, placed downstream of a furin cleavage site, designed to remove peptide overhangs on the C-terminus of the upstream encoded polypeptide. (**C**) Representative example in which healthy donor T-cells were transduced with a retroviral vector encoding for CAR +/− chemokine receptor. After culture for 12 days in IL-2, cells were analyzed by flow cytometry for expression of the myc epitope-tagged CAR and indicated chemokine receptors by flow cytometry. SSC: side scatter. Quadrants were set using untransduced T-cells cultured in IL-2. Data are representative of replicate experiments undertaken with 7 independent donors.

We have previously described A20-28z, which is a CD28+CD3ζ-containing second generation CAR that binds the αvβ6 integrin [15]. Specificity of targeting is achieved using a 20-mer peptide (A20) derived from a viral capsid protein of foot and mouth disease virus. To generate a matched control peptide (C20), A20 was mutated at the key arginine-glycine-aspartic acid-leucine (RGDL) integrin binding domain, each of which was substituted by alanine (Figure 2A). Given that several αvβ6-positive tumor cell lines were found to secrete IL-8 both in vitro and when grown as xenografts in vivo, we engineered A20-28z CAR T-cells to co-express the IL-8-specific chemokine receptors, CXCR1, or CXCR2 (Figure 2B). Comparable expression of both transgene products was determined by flow cytometric analysis of human T-cells transduced with either A20-28z CXCR1 or A20-28z CXCR2 (Figure 2C). In contrast, neither CXCR1 nor CXCR2 was detected on the surface of untransduced T-cells or T-cells transduced with the A20-28z CAR (Figure 2C).

**Figure 3:**
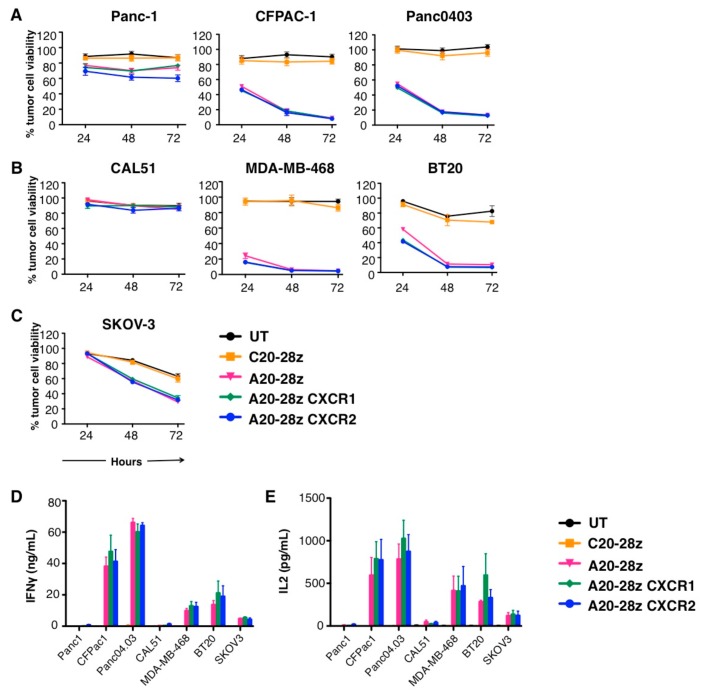
In vitro assessment of anti-tumor activity of chemokine receptor-expressing CAR T-cells targeted against αvβ6. Pancreatic (**A**), triple negative breast (**B**), or ovarian (**C**) tumor cells were co-cultivated at a 1:1 ratio with the indicated CAR T-cells in the absence of exogenous cytokine for 24–72 h. Data show the mean ± SEM of residual tumor cell viability from 3 independent experiments, each performed in triplicate and quantified by MTT assay. At each time-point, percentage tumor cell survival has been expressed relative to untreated tumor cells (set at 100% viability). Supernatant was collected and levels of IFN-γ (48 h; **D**) and IL-2 (24 h; **E**) quantified by ELISA. Data show the mean ± SEM of cytokine concentration detected in 3 independent experiments.

To assess whether co-expression of CXCR1 or CXCR2 affected the anti-tumor activity of A20-28z^+^ CAR T-cells, co-cultures were established between αvβ6-positive tumor cells that secrete IL-8 (Figure 1A) and CAR T-cells. Tumor cell-killing was quantified by MTT (3-(4,5-Dimethylthiazol-2-yl)-2,5-Diphenyltetrazolium Bromide) assay (Figure 3A–C). No killing of αvβ6-negative CAL51 or Panc1 tumor cells was observed upon co-culture with A20-28z, A20-28z or A20-28z CXCR2 CAR T-cells (Figure 3A,B). Killing of αvβ6-positive pancreatic (Figure 3A), breast (Figure 3B) or ovarian (Figure 3C) tumor cells and secretion of either IFN-γ (Figure 3D) or IL-2 (Figure 3E) was comparable after co-culture with A20-28z^+^ CAR T-cells, irrespective of whether they co-expressed an IL-8-responsive chemokine receptor or not. Together, these data indicate that inclusion of a chemokine receptor does not alter target-dependent specificity or activation of CAR T-cells in vitro. 

### 2.3. A20-28z CXCR2 CAR T-Cells Migrate More Efficiently towards IL-8

**Figure 4:**
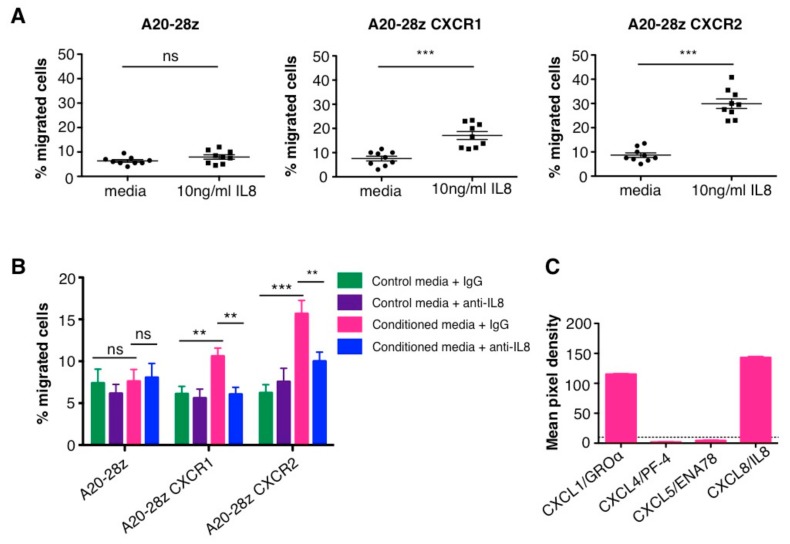
In vitro migration of chemokine receptor CAR T-cells to IL-8. (**A**) Media or media containing 10 ng/mL recombinant human IL-8 was added to the lower well of a transwell migration plate. CAR T-cells were added to the upper chamber and the percentage of migrated T-cells in the lower well was quantified after 2 h. Data show the percentage of migrated cells in triplicate wells from 3 independent experiments (mean ± SEM); *** *p* < 0.0001, calculated using Student’s *t*-test. (**B**) Conditioned media from SKOV3 cells that contained either an IgG control or an anti-IL-8 blocking antibody was added to the lower well of a transwell migration plate. CAR T-cells were added to the upper chamber and the percentage of migrated T-cells in the lower well was quantified after 2 h. Data show the percentage of migrated cells within triplicate wells from 3 independent experiments (mean ± SEM); ns: not significant; ** *p* < 0.01; *** *p* = 0.0001 calculated using an unpaired, two-tailed Student’s *t*-test. (**C**) Secretion of CXCL1 and IL-8 (CXCL8) by SKOV3 cells. Supernatant from SKOV3 was applied to nitrocellulose membranes spotted with capture antibodies and then detected using biotinylated antibodies followed by chemiluminescent detection reagents. Mean pixel density from duplicate wells was calculated using Image J software.

To test the functionality of CXCR1 or CXCR2, we investigated the ability of A20-28z CXCR1/2 CAR T-cells to migrate towards recombinant human IL-8 when placed on the other side of a transwell. The mean basal level of migration of A20-28z^+^ T-cells to media alone was 6% over 2 h (range 4–9.5%) and this did not change significantly when 10 ng/ml IL-8 was added to the lower chamber (mean 8%). In contrast, co-expression of CXCR1 with A20-28z resulted in over a twofold increase in the number of cells migrating towards IL-8 (mean 17%; range 12–23%). Co-expression of CXCR2 further increased the number of CAR T-cells migrating to IL-8 to 30% (range 23–41%) (Figure 4A). 

Similarly, both A20-28z CXCR1^+^ and A20-28z CXCR2^+^ CAR T-cells migrated more efficiently towards conditioned media from SKOV3 cells compared to A20-28z^+^ CAR T-cells (Figure 4B). To demonstrate that this increase in migration was due to IL-8 rather than other chemokines in conditioned media, the same experiment was performed in the presence of an IL-8 blocking antibody. This completely abrogated the enhanced migration of A20-28z CXCR1^+^ CAR T-cells and significantly (but not fully) decreased migration of A20-28z CXCR2^+^ CAR T-cells. This may reflect the ability of CXCR2 to bind other ELR-positive chemokines such as CXCL1/GROα, which is also secreted by SKOV3 cells (Figure 4C). 

Although both chemokine receptors demonstrated functional activity, CXCR2 promoted superior IL-8-dependent migratory properties compared to CXCR1 and was advanced for further in vivo testing.

### 2.4. A20-28z CXCR2 Have Improved Therapeutic Efficacy against Pancreatic and Ovarian Xenograft Models

**Figure 5:**
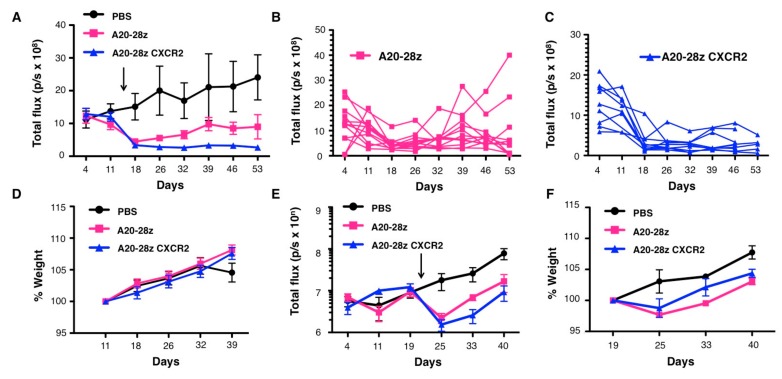
Improved therapeutic efficacy of chemokine-responsive CAR T-cells in vivo. (**A**) Mice were injected using the intraperitoneal (IP) route with 2 × 10^6^ Panc04.03-firefly luciferase cells and tumors were allowed to establish for 14 d before IP treatment with a single dose of 2 × 10^7^ of the indicated gene-modified T-cells. The arrow indicates the day of treatment with CAR T-cells. Control mice received phosphate buffered saline (PBS). Bioluminescence imaging using d-luciferin (substrate for ffluc) was used to monitor tumor status. Data show the mean ± SEM of tumor-derived total flux of 9–12 mice per group pooled from two independent experiments. * *p* < 0.05 comparing A20-28z vs. A20-28z CXCR2 (unpaired, two-tailed Student’s *t*-test). Bioluminescence emission from A20-28z (**B**) and A20-28z CXCR2-treated mice (**C**) are also shown. (**D**) Weight of treated mice relative to pre-treatment weight (mean ± SEM pooled from 2 independent experiments). (**E**) Mice were injected IP with 1 × 10^6^ SKOV3-firefly luciferase cells and tumor burden monitored thereafter using bioluminescence imaging. After 21 days, mice were treated with a single dose of 2 × 10^7^ of the indicated gene-modified T-cells (arrowed). Control mice received PBS. Tumor burden and weight (expressed relative to pre-treatment weight; **F**) was monitored weekly (mean ± SEM, *n* = 5 mice per group).

In order to evaluate the therapeutic activity of A20-28z CXCR2^+^ T-cells in vivo, three tumor xenograft models were established that co-express αvβ6 and IL-8. A single dose of A20-28z^+^ CAR T-cells resulted in regression of Panc0403 tumors (Figure 5A). Stable disease was achieved in the majority of mice although relapse was subsequently evident in 2/12 mice prior to the experimental endpoint (Figure 5B). In contrast, although A20-28z CXCR2^+^ CAR T-cell treated mice had similar initial response rates at day 18, tumor control was significantly improved from day 26 onwards compared to A20-28z-treated mice. All A20-28z CXCR2^+^ CAR T-cell treated mice had stable low burden tumor at the experimental endpoint (Figure 5C). Although the A20 peptide binds to mouse αvβ6 integrin, the enhanced therapeutic efficacy of A20-28z CXCR2^+^ CAR T-cells was not associated with clinically evident toxicity or weight loss (Figure 5D). A trend towards improved efficacy of A20-28z CXCR2 compared to A20-28z was also observed against an advanced SKOV3 xenograft (Figure 5E) in the absence of any weight loss (Figure 5F). 

**Figure 6:**
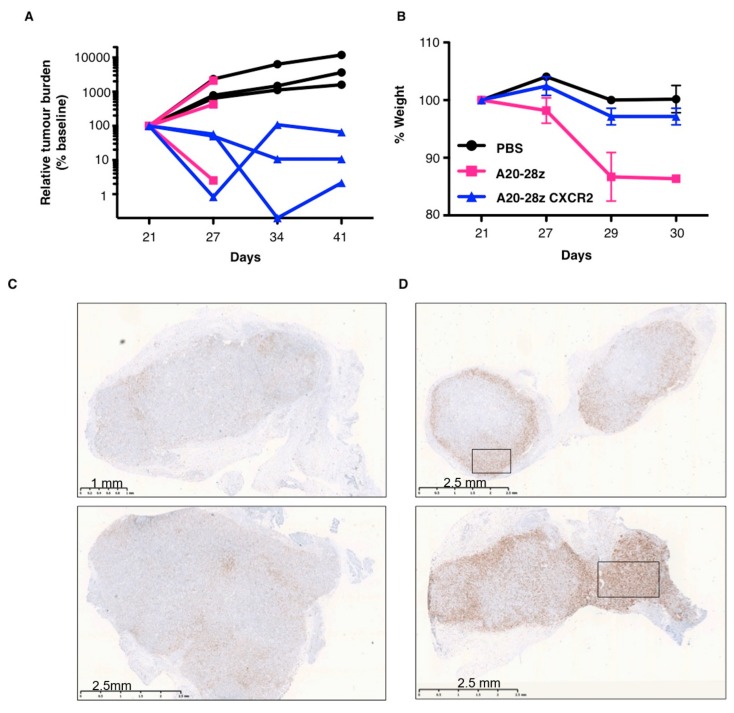

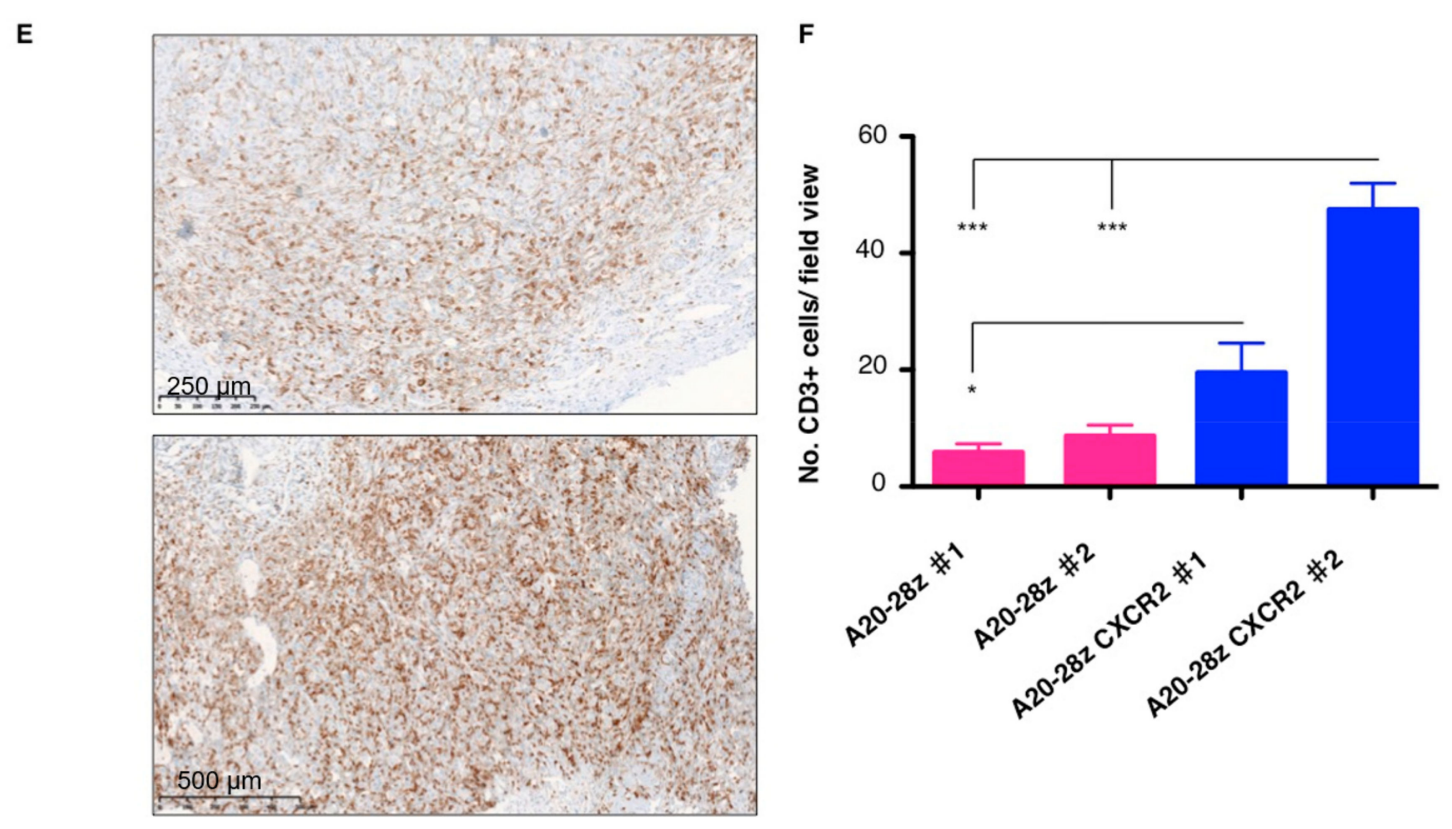
Improved efficacy, tumor-homing and safety of A20-28z CXCR2 T-cells. (**A**) Mice were injected subcutaneously with 1 × 10^6^ CFPac1-firefly luciferase cells and tumors were allowed to establish before treatment with a single dose of 5 × 10^6^ CAR^+^ T-cells, delivered IV on day 22. Control mice received PBS. Bioluminescence imaging using d-luciferin (substrate for ffluc) was used to monitor tumor status. Data show the tumor-derived total flux of individual mice. (**B**) Weight of treated mice relative to pre-treatment weight (mean ± SEM; *n* = 3 mice per group). Tumors were harvested from mice treated with A20-28z (**C**) or A20-28z CXCR2 CAR T-cells (**D**) and stained for the presence of T-cells using an anti-human CD3 antibody. Higher magnification images of boxed regions in D are shown in (**E**). (**F**) Quantification of CD3 staining. Data shows the mean ± SEM number of CD3+ve cells per field of vision from 13–20 high magnification (×40) fields of vision per tumor section shown in (**C**) and (**D**).

To further test the safety of A20-28z CXCR2, CAR T-cells were delivered intravenously to mice with a CFPac1 subcutaneous xenograft (Figure 6A). Tumor regression was observed in all three treated mice, with all animals having a lower tumor burden at the experimental endpoint than was observed pre-treatment. In contrast, only one of three mice treated with A20-28z CAR T-cells that lack CXCR2 showed an anti-tumor response with the remainder developing progressive disease, comparable to that seen in control mice. This was despite comparable activity of CAR T-cells in vitro (Figure S1). Unexpectedly, treatment with A20-28z^+^ CAR T-cells led to significant weight loss in all mice that exceeded humane endpoints and required euthanasia on day 31. In contrast, weight loss was not observed in mice treated with A20-28z CXCR2^+^ T-cells, similar to control-treated mice (Figure 6B). A similar pattern of weight loss was also observed following intravenous (IV) delivery of A20-28z^+^ CAR T-cells to a SKOV3 xenograft (data not shown). To investigate homing of T-cells, tumor tissue was harvested and stained for the presence of human CD3 following treatment with either A20-28z (Figure 6C) or A20-28z CXCR2 (Figure 6D,E). Limited T-cell infiltration was observed in mice treated with A20-28z and this tended to be restricted to the tumor periphery. In contrast, a significantly stronger T-cell presence was observed in the tumors of two mice treated with A20-28z CXCR2 CAR T-cells (the third mouse had no macroscopically evident tumor at the experimental endpoint), including infiltration into the tumor core (Figure 6D,F).

## 3. Discussion

Despite spectacular clinical success in B-cell malignancy, the majority of patients with solid tumors do not benefit in a meaningful way following CAR T-cell immunotherapy [39,40,41,42]. The paucity of tumor-specific antigens is a major obstacle in this regard. Furthermore, intravenous delivery is required for treatment of metastatic disease. This means that vital organs such as lungs and liver are highly exposed to these cells, favoring the occurrence of on-target off-tumor toxicity as a result of CAR T-cell recognition of low levels of antigen [43,44]. In contrast, only a small proportion of infused CAR T-cells successfully reach tumor sites [45]. These considerations suggest that development of strategies to improve CAR T-cell homing to sites affected by cancer might have the twin advantages of improving therapeutic efficacy while reducing potential for toxicity to normal tissues.

Aberrant expression of the epithelial-specific integrin αvβ6 is prevalent in many solid tumors [1,2,3,4,5,6,7,8]. In contrast, this integrin is scarcely detectable in normal healthy tissue besides weak/sporadic expression in basal cells of squamous epithelium, along the colonic columnar epithelium and in the placenta [2,10,46], rendering it an attractive clinical candidate target. A humanized mAb targeting αvβ6 has been tested in Phase 2 trials against idiopathic pulmonary fibrosis (NCT01371305) although safety and efficacy data has not yet been reported. However, pre-clinical studies have demonstrated no toxicity of αvβ6-targeted antibodies [3,13,46] and late-stage clinical trials using the pan-αv antibodies abituzumab and intetumumab against colorectal cancer and melanoma have demonstrated favorable safety profiles [47]. Nonetheless, given that CAR T-cells can respond to levels of antigen lower than those required for a response to antibody treatment [48], on-target off-tumor toxicity remains a concern for all potential targets in CAR T-cell immunotherapy. We have previously shown that CAR T-cells targeting αvβ6 can control tumor growth in xenograft models of pancreatic, breast, and ovarian cancer [49]. We utilized the small peptide A20FMDV2 as a targeting moiety, exploiting the fact that it is between 85–1000-fold more specific for αvβ6 over other integrins [50,51]. Accordingly, we previously found that A20-28z^+^ CAR T-cells elicited none or tolerable toxicity [15], in mouse models that can robustly recapitulate cytokine release syndrome [52].

Here, we have combined the A20-28z CAR with an approach designed to overcome inefficient homing of CAR T-cells to malignant lesions, exploiting the high levels of IL-8 secreted by numerous tumor types [53,54]. T-cells do not express the IL-8-responsive chemokine receptors, CXCR1 or CXCR2, and consequently they are unable to respond naturally to a tumor-derived gradient of IL-8. Nonetheless, it has been demonstrated that ectopic expression of CXCR2 in T-cells from normal donors or tumor-associated lymphocytes from ovarian cancer patients can increase their migration towards recombinant IL-8 and autologous ascites that contains CXCL1 and CXCL8 (IL-8) [30,54]. Similarly, expression of CXCR2 in transgenic pmel-1 or MAGE-A3 T-cell receptor-engineered T-cells enhanced their ability to migrate to subcutaneous xenografts of melanoma and colonic origin [55,56]. Others have demonstrated feasibility of such an approach in the context of CAR T-cell immunotherapy by co-expression of the chemokine receptors CCR2 or CCR4 in these cells [57,58].

Here, we have demonstrated that IL-8 is produced by a range of αvβ6-positive pancreatic, ovarian, and triple-negative breast cancer cell types and derived xenografts. Using these models, we found that co-expression of CXCR1 and CXCR2 with an αvβ6-specific CAR resulted in a significant increase in T-cell migration towards recombinant or tumor-derived IL-8, without alteration of CAR-mediated cytolytic activity or cytokine release. Although CXCR2 has a lower affinity for IL-8 than CXCR1, it proved more potent in this respect. This may reflect the promiscuity of CXCR2, enabling it to also respond to other tumor-derived ELR chemokine ligands, such as CXCL1/GROα. We went on to demonstrate that T-cells which co-express A20-28z and CXCR2 achieve enhanced tumor control in advanced pancreatic and ovarian tumor xenograft models. The A20FMDV2 peptide crosses the species barrier and binds strongly to the mouse ortholog of αvβ6 integrin [15]. Nonetheless, although we used a mouse model that readily allows the detection of CAR T-cell induced cytokine release syndrome [52], no toxicity was observed following the administration of A20-28z CXCR2^+^ CAR T-cells, even after intravenous delivery. Indeed, it is noteworthy that in these experiments, weight was stable in A20-28z CXCR2^+^ CAR T-cell-treated mice in contrast to control animals that received A20-28z^+^ CAR T-cells. Although untested, this raises the possibility that IL-8 was able to preferentially attract T-cells to the tumor rather than to other organs where toxicity could ensue. In support of this, more T-cells were observed in the tumors of A20-28z CXCR2^+^ CAR T-cell-treated mice compared to mice treated with A20-28z^+^ CAR T-cells. 

In short, these data show that CAR T-cell immunotherapy can be made more effective through expression of a receptor that matches the chemokine milieu present in the tumor microenvironment. Given the prevalence of IL-8 production in human cancer and its link to tumor-associated hypoxia [59], this approach may find broad applicability in the potentiation of CAR T-cell immunotherapy for solid tumors.

## 4. Materials and Methods 

### 4.1. Retroviral Constructs

The A20-28z and C20-28z retroviral constructs have been described previously [15]. Briefly, the VP1-derived A20FMDV2 20mer peptide (termed A20) was placed downstream of a CD124 signal peptide selected for correct predicted cleavage site using SignalP 3.0 server [60] (Genscript, Piscataway, NJ, USA). A control αvβ6 non-binding peptide (termed C20) was generated in which the RGDL motif within A20FMDV2 was substituted with AAAA (Figure 2A). To enable detection of CARs, a myc epitope-tagged framework was engineered (Figure 2B). A codon-optimized cDNA was synthesized to encode for human CD28 (amino acids 114 to 200) in which B7-binding residues 117–122 (MYPPPY) were substituted with residues 410–419 of human c-myc (EQKLISEEDL) (Mr Gene, Regensburg, Germany). The sequence was flanked by 5′ Not1 and 3′ Apa1 restriction sites and was substituted for the corresponding fragment in SFG T1E28z, (encodes a broadly ErbB reactive CAR with a CD28+CD3ζ endodomain) [61]. A20 and C20 cDNA sequences were cloned into SFG-Tm28z at the NcoI/NotI restriction enzyme sites (Figure 2B). 

In order to co-express the human chemokine receptor CXCR1 or CXCR2, codon-optimized cDNAs flanked by NcoI restriction enzyme sites were cloned into the A20-28z retroviral construct. Stoichiometric co-expression was achieved using an intervening furin cleavage site (RRKR), [serine-glycine]_2_ linker and *Thosea Asigna* 2A (T2A) peptide (Figure 2B). 

To visualize tumors in vivo, firefly luciferase (ffluc) was co-expressed with tdTomato red fluorescent protein (tdRFP; Genscript) in a single SFG retroviral vector containing a furin cleavage site and T2A peptide (SFG ffluc/tdTom).

### 4.2. Culture and Retroviral Transduction of Primary Human T-Cells

Blood samples were obtained from healthy volunteers with approval of the South East London Research Ethics Committee 1 (reference 09/H0804/92). Activation of T-cells was achieved 48 h prior to gene transfer using CD3+CD28-coated paramagnetic beads (1:1 bead: cell ratio; Thermo Fisher Scientific, Paisley, UK). Gene transfer was performed using PG13 retroviral packaging cells. Transduced T-cells were cultured in RPMI-1640 supplemented with 10% human AB serum (Sigma, Poole, UK), GlutaMax and antibiotic-antimycotic solution (Thermo Fisher Scientific, Paisley, UK) and in the presence of 100 U/mL IL-2 (Proleukin, Novartis, Frimley, UK). T-cells were typically cultured for 11–12 days prior to use in experiments. 

### 4.3. Cell Lines

Firefly luciferase positive (ffluc^+^) Panc-1, BxPC3, CFPAC1, and Panc0403 pancreatic cancer cells were obtained from the Barts Cancer Institute, Queen Mary University of London. Ffluc^+^ SKOV-3 ovarian cancer cells were purchased from Caliper (PerkinElmer, Waltham, MA, USA). The breast cancer cell lines MCF7, T47D, BT474, ZR75-1, CAL51, BT20, and MDA-MB-468 were obtained from the Breast Cancer Now Research Unit, King’s College London. Tumor cell lines were grown in R10 or D10 medium, respectively comprising RPMI or DMEM (Lonza, Basel, Switzerland) supplemented with 10% FBS (Sigma, Poole, UK), GlutaMax, and antibiotic-antimycotic solution (Thermo Fisher Scientific, Paisley, UK). PG13 retroviral packaging cells were obtained from the European Collection of Cell Cultures (ECACC, Porton Down, UK) and were maintained in D10. H29 retroviral packaging cells were a gift from Dr. Michel Sadelain (Memorial Sloan Kettering Cancer Center, New York, NY, USA). 

### 4.4. Flow Cytometry Analysis

Expression of CARs was detected using supernatant derived from the 9e10 hybridoma (ECACC), which binds to residues 410–419 of human c-myc, followed by goat anti-mouse Ig-PE. Untransduced T-cells acted as a negative control. Chemokine receptors were detected using either a FITC-conjugated anti-CXCR1 antibody (Biolegend 320605, London, UK) or a PR-conjugated anti-CXCR2 antibody (Biolegend 320705, London, UK). Flow cytometry was performed using a FACSCalibur cytometer with CellQuest Pro software or BD LSRFortessa cytometer with BD FACSDiva software (all BD Biosciences, Wokingham, UK).

### 4.5. Cytotoxicity Assays

Tumor cells were seeded at a density of 1 × 10^4^ cells/well in a 96 well plate and incubated with T-cells for 24–72 h at a 1:1 tumor cell: T-cell ratio or as otherwise specified. Destruction of tumor cell monolayers by T-cells was quantified using an MTT assay. MTT (Sigma, Poole, UK) was added at 500 µg/mL in D10 medium for 2 h at 37 °C and 5% CO_2_. After removal of the supernatant, formazan crystals were resuspended in 100 μL DMSO. Absorbance was measured at 560 nm. Tumor cell viability was calculated as (absorbance or luminescence of monolayer cultured with T-cells/absorbance or luminescence of untreated monolayer alone) × 100%.

### 4.6. Protein Analysis

Supernatants collected from co-culture of tumor cells with CAR T-cells was analyzed using a human IFN-γ or human IL-2 enzyme-linked immunosorbent assay (ELISA) Ready-set-go kit (eBiosciences, Hatfield, UK), as described by the manufacturers. Tumor cell supernatant or plasma from mice was analysed using a human IL-8 ELISA kit (ThermoFisher 88-8086-88, Paisley, UK). For tumor cell supernatant, cells were seeded at a density of 5 × 10^5^ cells/well/2 mL media in a 6-well plate and supernatant harvested 48 h later. To detect ELR chemokines secreted from SKOV3 cells, a Proteome profiler human XL cytokine array kit was used according to the manufacturers’ instructions (Bio-Techne ARY022B, Abingdon, UK).

### 4.7. T-Cell Migration Assay 

T-cell migration assays were performed using 24-well transwell migration plates with a 6.5 mm insert and 5 μM pores (Corning, 3421). Either 10 ng/mL recombinant human IL-8 (BioTechne 208-IL, Abingdon, UK) or conditioned media from SKOV-3 cells (overnight culture in 0.1% FBS DMEM) was added in 500 μL RPMI to the lower chamber and 5 × 10^5^ T-cells in 200 μL media to the upper chamber. T-cell migration to the bottom chamber was quantified after 2 h by counting the number of migrated cells from triplicate wells. In some experiments, using conditioned media, an anti-hIL-8 blocking antibody (BioTechne MAB208, Abingdon, UK) or mouse IgG1 isotype control (Biolegend clone MOPC-21 400101, London, UK), was included in the bottom chamber at 2.5 μg/mL. 

### 4.8. In Vivo Studies 

All in vivo experimentation adhered to U.K. Home Office guidelines, as specified in project license number 70/7794 and was approved by the King’s College London animal welfare and ethical review body (AWERB) on March 28th, 2018. Where necessary, tumor cells were transduced with SFG ffluc/tdTom and were purified by flow sorting prior to engraftment in vivo (MDA-MB-468 and Bxpc3 cells only). Intraperitoneal tumor models were established in SCID-Beige mice following inoculation with either SKOV-3, CFPac-1, Bxpc3 or MDA-MB-468 cells (1 × 10^6^ cells each), or Panc0403 cells (2 × 10^6^ cells). Orthotopic tumors were established by injection of 1 × 10^6^ Panc0403 cells and Matrigel (Corning) in a final volume of 25 μL using a Hamilton syringe (Sigma, Poole, UK), directly into the pancreas under sterile surgical conditions. Subcutaneous CFPac1 were established in NSG mice by injection with 1 × 10^6^ cells.

To assess levels of circulating human IL-8 in tumor-bearing mice, blood was collected by cardiac puncture into Eppendorf tubes containing citrate-dextrose (Sigma, Poole, UK) and centrifuged at maximum speed for 10 min at 4 °C in a microcentrifuge. 

In experiments establishing the safety and therapeutic efficacy of CAR T-cells, engineered T-cells (2 × 10^7^ cells in total) were administered IP on day 14 (Panc0403) or day 21 (SKOV-3). Where CAR T-cells were delivered intravenously, a single dose of 5 × 10^6^ cells CAR^+^ T-cells was injected intravenously on day 22 to mice bearing CFPac1 subcutaneous tumors. CFPac1 tumors from mice treated with A20-28z and A20-28z CXCR2 were harvested on days 31 and 41, respectively. 

Bioluminescence imaging (BLI) was performed using an IVIS Spectrum Imaging platform (PerkinElmer) with Living Image software (PerkinElmer, Beaconsfield, UK). To monitor tumor status, mice were injected IP with D-luciferin (150 mg/kg; PerkinElmer, Beaconsfield, UK) and imaged under isoflurane anesthesia after 12 min. Magnetic resonance imaging (MRI) was performed using a Bruker Icon 1T MRI scanner to mice bearing Panc0403 orthotopic tumors that had been allowed to establish for 46 d. In all experiments, animals were inspected daily and weighed weekly.

### 4.9. Histology

Mouse tissue was fixed in 10% formalin (4% formaldehyde) for 24 h and then transferred to 70% ethanol. Organs were paraffin embedded and 4 μm slices sectioned before staining with hematoxylin and eosin (H and E). Sections were stained using a rabbit anti-human CD3 antibody (Agilent Technologies, Cheshire, UK) at a 1:300 dilution. Processing and staining were performed by the Pathology Services at Barts Cancer Institute, QMUL. Quantification of CD3 staining was performed manually by counting the number of CD3+ve cells per high magnification (40×) field of view. 13–20 fields of vision were taken to span the largest diameter of each tumor section. Each field was approximately 150 × 250 μm. The number of CD3+ve cells per field was then counted and the mean±SEM calculated. 

### 4.10. Statistical Analysis

For comparison of the two groups, datasets were analyzed using two-tailed Student’s *t*-test. All statistical analysis was performed using GraphPad Prism version 5.0 or 6.0 (GraphPad software, San Diego, CA, USA) or Excel for Mac 2011 (Microsoft, Berkshire, UK). 

## 5. Conclusions

We demonstrate that IL-8 is secreted by a range of tumor cell lines both in vitro and when grown as xenografts in immunocompromised mice. Co-expression of the matched chemokine receptor CXCR2 in 2nd generation CAR T-cells targeted to the integrin αvβ6 results in increased migration in response to recombinant hIL-8 and to tumor-cell derived supernatant in an IL-8-mediated manner, without affecting cytotoxic function. A20-28z CXCR2 T-cells demonstrate superior anti-tumor activity in vivo against αvβ6-expressing tumor xenografts and, although the CAR also engages the mouse ortholog of αvβ6, therapy was well tolerated. Given the frequency at which αvβ6 is overexpressed in solid tumors and the prevalence of IL-8 production in the tumor microenvironment, this provides a strong rationale for combining highly tumor-specific CAR T-cells with chemokine receptors to improve homing and therapeutic activity.

## Supplementary Materials

The following are available online at https://www.mdpi.com/2072-6694/11/5/674/s1, Figure S1: (A) Transduction of T-cells used for homing to a CFPac1 subcutaneous xenograft. Healthy donor T-cells were transduced with a retroviral vector encoding for CAR +/− chemokine receptor. (B) In vitro cytotoxicity of CAR T-cells prior to intravenous injection into mice. CAR T-cells were co-cultured with Bxpc3 pancreatic tumour cells at varying effector:target ratios in the absence of exogenous cytokine for 72 h. Data show the mean ± SEM of residual tumour cell viability from a single experiment performed in triplicate and quantified by MTT assay.
