# Supplementary material for: CAR T-Cells Targeting the Integrin αvβ6 and Co-Expressing the Chemokine Receptor CXCR2 Demonstrate Enhanced Homing and Efficacy against Several Solid Malignancies
Source: Cancers (Basel). 2019 May 14;11(5):674. doi: 10.3390/cancers11050674 (PMC6563120; doi:10.3390/cancers11050674)
Supplement: Supplementary file 1 [file cancers-11-00674-s001.pdf]

Supplementary Materials: CAR T-Cells Targeting the Integrin αvβ6 and Co-Expressing the Chemokine Receptor CXCR2 Demonstrate Enhanced Homing and Efficacy Against Several Solid Malignancies

Lynsey M. Whilding, Leena Halim, Ben Draper, Ana C. Parente-Pereira, Tomasz Zabinski, David M. Davies and John Maher

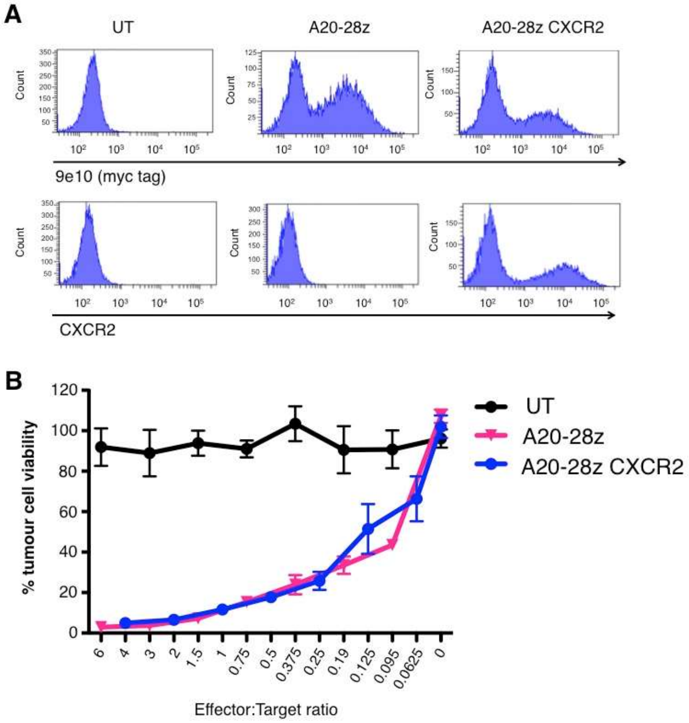

Figure S1. (**A**) Transduction of T-cells used for homing to a CFPac1 subcutaneous xenograft. Healthy donor T-cells were transduced with a retroviral vector encoding for CAR +/− chemokine receptor. After culture for 12 days in IL-2, cells were analysed by flow cytometry for expression of the myc epitope-tagged CAR and CXCR2 by flow cytometry. (**B**) In vitro cytotoxicity of CAR T-cells prior to intravenous injection into mice. CAR T-cells were co-cultured with Bxpc3 pancreatic tumour cells at varying effector:target ratios in the absence of exogenous cytokine for 72 h. Data show the mean ± SEM of residual tumour cell viability from a single experiment performed in triplicate and quantified by MTT assay.

© 2019 by the authors. Licensee MDPI, Basel, Switzerland. This article is an open access article distributed under the terms and conditions of the Creative Commons Attribution (CC BY) license (http://creativecommons.org/licenses/by/4.0/).
